# Frequency, Time, Representation and Modeling Aspects for Major Speech and Audio Processing Applications

**DOI:** 10.3390/s22166304

**Published:** 2022-08-22

**Authors:** Juraj Kacur, Boris Puterka, Jarmila Pavlovicova, Milos Oravec

**Affiliations:** 1 Institute of Multimedia Information and Communication Technologies, Faculty of Electrical Engineering and Information Technology STU, 812 19 Bratislava, Slovakia; 2 Institute of Robotics and Cybernetics, Faculty of Electrical Engineering and Information Technology STU, 812 19 Bratislava, Slovakia; 3 Institute of Computer Science and Mathematics, Faculty of Electrical Engineering and Information Technology STU, 812 19 Bratislava, Slovakia

**Keywords:** speech features, speaker recognition, speech recognition, speech emotions, audio event recognition, convolutional neural networks

## Abstract

There are many speech and audio processing applications and their number is growing. They may cover a wide range of tasks, each having different requirements on the processed speech or audio signals and, therefore, indirectly, on the audio sensors as well. This article reports on tests and evaluation of the effect of basic physical properties of speech and audio signals on the recognition accuracy of major speech/audio processing applications, i.e., speech recognition, speaker recognition, speech emotion recognition, and audio event recognition. A particular focus is on frequency ranges, time intervals, a precision of representation (quantization), and complexities of models suitable for each class of applications. Using domain-specific datasets, eligible feature extraction methods and complex neural network models, it was possible to test and evaluate the effect of basic speech and audio signal properties on the achieved accuracies for each group of applications. The tests confirmed that the basic parameters do affect the overall performance and, moreover, this effect is domain-dependent. Therefore, accurate knowledge of the extent of these effects can be valuable for system designers when selecting appropriate hardware, sensors, architecture, and software for a particular application, especially in the case of limited resources.

## 1. Introduction

The number of various speech and audio applications is growing with the rise of artificial intelligence, machine learning, and suitable hardware platforms. Such applications can be in a form of simple standalone applications, edge computing solutions or even huge cloud-based systems. Since both speech and audio signals convey a lot of very useful information, such systems cover a wide range of applications, e.g., speech recognition systems [1], speaker identification or verification systems [2], speaker diarization, speech emotion recognition [3], detection and recognition of events in audio signals [4], etc. These applications are deployed in different branches of industry, information systems, medicine, security systems, or just in everyday life. As a consequence, these very diverse applications differ in requirements for processed speech or audio signals, which sets requirements on the input audio sensors as well. Moreover, it is known the performance of such applications is affected by the quality, quantity, and representation of audio and speech signals. Therefore, knowing how the basic physical properties of speech and audio signals affect the performance of a particular group of applications is important in reducing complexity and cost while preserving high performance.

Based on this, we tested and assessed popular speech and audio processing applications, i.e., speech recognition, speaker recognition, speech emotion recognition, and audio event detection/recognition. We did it using a unified framework, providing multiple comprehensive results in the same place. The effect of basic physical properties of speech and audio signals, i.e., frequency ranges, time aspects, quantization (representation), and model complexity, on the performance of major applications was measured. To minimize the dependency of results on improperly selected speech/audio features and classification methods, a rather general signal representation was chosen using spectrograms and vocal tract features, e.g., [5]. Spectrograms provide vital time–frequency distributions while preserving most of the valuable information. In the modeling, convolutional neural networks (CNN) were used, as they currently provide state-of-the-art results.

The remainder of the article is organized as follows. First, a survey of related articles on speech, speaker, emotion, and audio event recognition systems is given. Next, the selected feature extraction and classification methods are briefly explained. In the practical part, a short description of four datasets is given together with basic settings common to all experiments. Finally, the results achieved are presented for each domain and are later discussed and summarized.

## 2. Related Work

As the article focuses on four major speech and audio processing applications, this section is divided into four subsections.

### 2.1. Speech Emotion Recognition

Speech emotion recognition systems (SER) are mostly used in call centers or in an online learning process [3]. They also have their place in medicine, e.g., detection of specific behavioral changes or in the automotive industry, e.g., analyzing in-car conversation for safety reasons [6].

The SER-related articles can be roughly divided into two main classes, i.e., neural network (NN) based and NN-free solutions. In [7], a three-layer CNN processing spectrograms was compared with the pre-trained Alexnet network. It was found that using a pre-trained CNN on a different dataset had no real effect on the recognition rate. In [8], different-than-square, i.e., rectangular, kernels were tested, and in [9] multi-convolutional kernels used across different layers were suggested. In [10], several low-level acoustic features, such as Mel frequency cepstral coefficients (MFCC), extended Geneva minimalistic acoustic parameter set and high-level acoustic features were used with different classifiers, such as K-nearest neighbor (KNN), random forest, and support vector machines (SVM). In [11], a SVM classification method was applied to a mixture of MFCC, linear prediction cepstral coefficients, shifted delta spectrum, and RASTA-PLP (perceptual linear prediction) features. More recently, there have been several articles applying popular end-to-end processing to SER domain; e.g., in [12] end-to-end embeddings were derived using residual NN and a triplet loss function that were further evaluated by a cosine distance. Finally, in [13] we provided a complex analysis of basic speech properties and feature extraction methods in SER systems using two datasets. For a thorough overview of current SER systems, see, e.g., [14,15].

### 2.2. Speaker Recognition

The speaker recognition term comprises several tasks, e.g., speaker identification, speaker verification, speaker diarization [16], etc. Such systems have direct application in forensic science [17], biometric-based security systems, automatic speech annotation systems, etc. Both identification and verification systems can work in text-independent or text-dependent scenarios depending on the application. Classical speaker recognition methods (stage-wise) extract acoustic and sometimes prosodic features that are usually modeled by Gaussian mixture models (GMM) that could be adapted from a universal background model, e.g., [18]. Then, the overall probability is calculated by applying all feature vectors (FV) to the model. More recent methods derive a single fixed length vector from each recording, e.g., a supervector [19] or i-vector (low-dimensional supervector with suppressed session and speaker variability) that is compared with others using, e.g., SVM, NN, or a properly chosen vector distance. With the rapid growth of complex NN models and the accessibility of huge databases, NN-based solutions are becoming more popular, e.g., [20,21]. Here, different structures of NNs can be used to calculate i-vectors that compared to GMM based i-vectors can reduce the error even by 30% [22]. Furthermore, NNs can be used to derive deep embeddings of utterances either using frame-based labeling (d-vectors) or utterance-based labels (x-vectors) [17,20]. Such vectors (i, d, x) are then subjected to classification, which usually involves discriminative methods, e.g., SVM, NN. With deep NN-based solutions, the most popular features are spectrograms [23], filter banks [24], and MFCCs [25], while the network structures vary from recurrent (long short-term memory—LSTM, gated recurrent unit), convolutional [26], dense (feed forward), to even combinations of networks. There is also a successful branch of end-to-end systems, e.g., [27] that, unlike the stage-wise systems, take two recordings at the input for comparison and directly produce a similarity measure. A detailed survey of problems and methods in the speaker recognition domain can be found in, e.g., [22].

### 2.3. Speech Recognition

Speech recognition systems greatly differ in their complexity, applications, and structures. The simplest are designed to recognize isolated words (commands), whereas the most complex can recognize natural speech. Therefore, there are several classes of such systems, e.g., small-size vocabulary (hundreds of words), middle-size, and large-size (hundreds of thousands), working in real time (delay approx. 1 s) or offline systems (multi-pass), speaker-dependent or independent, etc. Different modifications of hidden Markov models (HMM) [28] used to be state-of-the-art for continuous speech recognition systems requiring acoustic, language, and pronunciation models. Currently, there are more promising approaches including hybrid NN-HMM [29,30], sequence-to-sequence [31], or end-to-end [32] systems. NN-HMM models combine deep NNs (convolutional or recurrent-LSTM) and HMM technologies [33], where NN parts model FVs/space and HMM is used for time modeling. Sequence-to-sequence approaches are based on NN structures utilizing, e.g., recurrent networks (recurrent network transducer), attention mechanisms (attention-based encoder-decoder), or connectionist temporal classification [32] that directly realize a sequence-to-sequence mapping, e.g., FVs to words. Their big advantage is that such systems need neither pronunciation dictionaries nor language models, so this extra expertise is not necessary. Different sorts of end-to-end approaches are becoming very popular that can process even raw signals and output texts without the explicit use of FVs. However, such an appealing approach requires a huge amount of data, very complex models, long training times, etc. Thus, currently, most of the successfully deployed systems still use hybrid NN-HMM models. Speech recognition is one of the most researched areas of speech processing with many developments; thus, for a more detailed overview of speech recognition technology see, e.g., [1,32].

### 2.4. Audio Event Recognition

Audio event recognition (AER) is a part of audio context analysis and its main application is in surveillance [34], monitoring [35], assistance to handicapped people, wildlife protection, and monitoring [36], etc. Originally, AER systems extracted specific or standard speech features that might have been fused and reduced in dimensionality prior to the classification by GMM, SVM, etc. [37]. Recently, complex CNN structures have been used providing the best scores. In this case several different approaches can be identified, e.g., CNNs using 1D convolution layers [38], 2D convolution [39], well-known pre-trained networks, especially from the image recognition domain [40], etc. The 1D convolution models rely on machines to learn the optimal structure of filters (end-to-end processing). The 2D convolution systems are applied to spectrograms or knowledge-based filters, e.g., Mel, Bark (psychoacoustic domain) to capture further details or patterns. In the case of using complex pre-trained networks, such networks are applied to the extracted 2D features (transfer learning), and these are the systems providing state-of-the-art results.

## 3. Materials and Methods

### 3.1. Theoretical Background on Speech, Audio Signals, and Perception

Speech signals are very complex as they convey not only lexical information (words, grammar), but quite a lot of additional information such as who we are, in what physical, health, and mental condition we are, etc. Most such information is added to the speech signal rather unintentionally (biometry, learned behavior). Therefore, it is important to understand the speech production process that consists of four distinct steps. These steps are: idea (what and how to say it), language that expresses the idea by the means of grammar and vocabulary (this reflects education, social background, etc.), prosody that includes intonation, stress, pauses, etc., (learnt behavior, emotions, etc.), and finally the physical production of air vibrations that is related to physical parameters of individuals. Therefore, for speech recognition, the first two stages are important, whereas in speaker and emotion recognition, all four stages can be used.

Speech signals are non-stationary; however, in short intervals (10 to 30 ms) they can be regarded as rather stationary. Their useful frequency range differs, e.g., 300 Hz to 4 kHz for speech recognition and telephony, and for speaker and emotion recognition a wider band is beneficial (approx. 20 Hz to 8 kHz). Speech has intervals of periodic like signals that exhibit the presence of a fundamental frequency, ranging from 80 Hz to even 300 Hz, which is important for, e.g., gender, speaker, speech emotion recognition, etc. Mathematically, a speech signal is composed of an excitation signal (lungs, vocal cords) and an impulse response of vocal tract (oral and nasal cavities, tongue, lips, etc.). The frequency characteristic of the vocal tract is important for the perception of different sounds as it contains formant (resonant) frequencies.

When speech perception is considered, it was found in, e.g., [5] that the widths and locations of the formant frequencies and their number are relevant. The frequency resolution declines with the frequency and the intensity is perceived non-linearly (logarithm-like). On the other hand, a tilt of spectrum in the form X(ω)ω^α^, narrow band stop filtering, and frequencies outside the band given by the first and third formant frequency are perception-irrelevant.

Except for speech signals, there is a huge class of environmental sounds. These sounds have very diverse time, energy, and frequency characteristics that are difficult to describe. There are several sound classifications using different criteria, e.g., acoustic, physical or semantic similarities, however, they are not yet standardized. For example, as given in [41] these are: animals, objects, background, impulsive, natural, artificial, etc. From the perspective of a human auditory system, audible signals are within a 20 Hz to 20 kHz frequency range, which, however, may not still contain a necessary classification range for some air vibrations. Thus, when processing such signals, in addition to speech processing strategies, basic psychoacoustic principles are also used.

Since speech and audio signals contain a great variety of information, it is common and useful to first extract eligible features emphasizing the needed information while suppressing the remaining data. On the other hand, the growing number of end-to-end processing systems partly diminishes the role of knowledge-based features. This is because the founding theory of these features may not be fully known, and the underlying conditions are rarely met in practise. However, end-to-end processing requires very complex models, huge computational power, and an enormous volume of representative data that are difficult to achieve.

### 3.2. Selection of Methods

Considering the main objective of the article, which is to measure and evaluate the effect of basic signals’ properties on the performance of major speech/audio applications with a direct connection to the design of acoustic sensors, the following feature extraction and classification methods were selected. In the case of feature extraction, spectrograms and Mel filter banks were chosen. This was because these basic acoustic features provide a valuable time–frequency distribution inevitable for analyzing non-stationary audio signals such as speech and environmental sounds. Moreover, these are the least modifying features, i.e., introducing minimal artificial modifications to the signal, preserving most of the acoustic relevant information. Because of their construction, they allow us to have a direct control over all important physical parameters, e.g., frequency and time ranges that may not be always possible using more complex or even fused features, where more aspects can be affected by a single setting. This would make the analysis rather ambiguous. Moreover, spectrograms are used as an intermediate stage in the calculation of more complex features such as MFCC, PLP, etc. Finally, these features themselves proved to be valuable in many speech processing applications, especially in combination with deep learning, e.g., [13,23,42,43], where it is the task of deep learning to find more appropriate features’ embeddings. As spectrograms are 2D signals exhibiting natural location and resolution variability, i.e., containing various patterns at different time and frequency locations, the natural choice for block-wise speech/audio classification are CNNs. CNNs are state-of-the-art models in image processing and play an important role also in speech/audio applications, e.g., [21,26,44,45]. The block-wise processing allows us to avoid using additional models for long (continuous) time modeling, e.g., language models in speech recognition, etc. that do affect the overall performance. On the other hand, such models (not acoustical) are not related to signal properties relevant to acoustic sensors. Moreover, this enables us to use a unifying classification framework that is application-independent. Finally, well-known, complex, and pre-trained CNNs (in addition to custom-designed CNNs) were tested using the popular transfer learning. Thus, the following sections will briefly summarize the above methods.

### 3.3. Spectrograms

**Figure 1:**
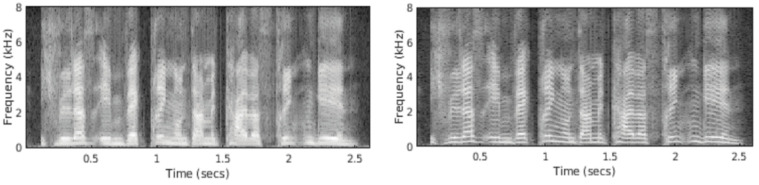
Spectrograms constructed using different window lengths; left 10 ms, right 30 ms.

Since one of the main objectives was to assess the effect of frequency ranges, spectrograms were the basic features in all experiments. Moreover, spectrograms are the least-processed common features that preserve vocal tract information, excitation signals, magnitudes, phases, etc., and many, more advanced features can be derived from spectrograms. This is vital as we did not want to introduce extensive modifications that could affect the results, and instead we let the complex NNs learn a proper representation of signals. Spectrograms represent a time–frequency distribution, and their key parameters are frame (windows) lengths used to calculate spectra by short-time Fourier transformation (STFT), window shapes, shifts between frames, and time spans of spectrograms. The frame lengths control a frequency resolution, i.e., the longer the frames, the higher the frequency resolution at the cost of losing time localization. This is documented in Figure 1 where 10 and 30 ms frames are applied. The frequency resolution is higher in the right figure, enabling us to see the course of fundamental frequency that is useful for, e.g., speaker and emotion recognition, prosody, etc. More on spectrograms, frequency resolution, and windows can be found in, e.g., [5].

### 3.4. Vocal Tract-Based Features—Filter Banks and Cepstral Coefficients

**Figure 2:**
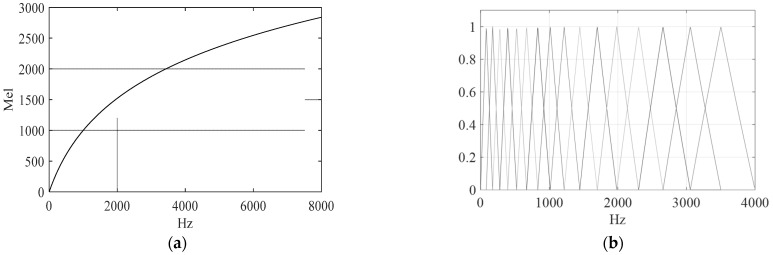
A mapping from hertz to Mel scale (**a**) and 16 Mel FBs for a 0–8 kHz frequency range (**b**).

In many applications where only the vocal tract information (spectral envelope and formant frequencies) is required, banks of filters are extracted. Then, a spectrum is divided into bands where each band is represented by an overall (weighted) intensity or power. This reduces the number of coefficients and may suppress noise while preserving relevant acoustic information. In audio processing, almost always auditory-based filter banks (FB) are used that follow some psychoacoustic phenomena. The most popular is Mel scale, which is related to the perception of a pure tone. A frequency mapping from 0–8 kHz to the Mel scale is shown in Figure 2a that prioritizes lower frequencies. The shape of Mel FBs (MFB) is shown in Figure 2b.

To make the representation of a spectral envelope even more compact, cepstral features can be derived. The most widely used are MFCCs [5] because of their good performance, compact representation, and easy computation. MFCCs are computed from Mel FBs by applying a logarithm and a DCT transformation. Logarithms are used not only as a part of cepstrum computation, but also mimic nonlinear sound perception. DCT II is applied to provide a good decorrelation within FVs and distributes energy only to a few components.

### 3.5. Quantization

Digital computers require quantization of signals; that is, a finite precision representation. This affects the quality of audio signals, requirements on the sensors, amount of processed data, transfer bit rates, etc. Quantization introduces quantization noise that may affect the performance of algorithms. In the case of uniformly distributed signals, the mean power of introduced noise (white) equals:

(1) $$P_{n}=\frac{1}{3}\frac{A_{max}^{2}}{2^{2bits}}$$

*A_max_* is a maximal amplitude, and *bits* is the number of quantization bits. For a standard CD quality, a linear 16 bit quantization is used. Due to nonlinear perception, especially in telephony, an 8 bit nonlinear (a-law, or μ-law) quantization is used. This means that the signal is nonlinearly compressed prior to quantization as follows (μ-law):

(2) $$X_{\mu }=\mathrm{sgn}\left(x\right)\frac{ln\left(1+255\left|x\right|\right)}{ln\left(256\right)},$$

where *X_μ_* and *x* are *μ*-law compressed and original signals, respectively. In the decoding phase, the signal is decompressed using the inverse of (2), i.e.,

(3) $$x=\mathrm{sgn}\left(X_{\mu }\right)\frac{256^{\left|X_{\mu }\right|}-1}{255}$$

This effectively helps to reduce bit rates while preserving acceptable quality.

### 3.6. Convolutional Neural Networks

Neural networks (NN) [46] are a favorable choice in many domains, as they can be complex, providing good generalization and robustness, while supporting adaptability and parallel computation. In particular, CNNs [47] and their modifications are the most successful in image and speech processing, because they can efficiently handle natural changes in resolution or location, while extracting finer patterns in a hierarchical order. Therefore, CNNs were the natural choice in this study to process spectrograms or FBs that are affected by variations in a similar way to pictures (except rotations).

CNNs use convolutional layers that filter an input with proper kernels, producing feature maps. Usually, such feature maps are analyzed and down-sampled by pooling layers, realizing the shift invariance. In majority of cases, the output layer of a CNN is a dense network realizing the final classification. In residual networks [48] some layers can be bypassed in a controlled manner, allowing for the existence of residual signals, and suppressing the problem of vanishing gradients. There are already several well-known complex pre-trained nets that are successfully applied even to different domains (transfer learning), e.g., Alexnet [49], Inception net [50], VGG [51], etc. Except in networks, there are many training algorithms that are mostly based on error back propagation, and the more advanced ones like ADAM automatically control weight updates using a gathered training statistic. Despite that, regularization techniques like Dropout and the introduction of training, testing, and validation sets with an early stopping criteria are deployed to secure a good and fast generalization.

## 4. Experimental Settings and Datasets

### 4.1. Software and Hardware

All experiments were performed using the unifying Python (3.8) environment. The signal processing part, including speech manipulation, spectrum calculation, and feature extraction, was performed using scipy, Librosa, and pydub packages. In the classification and modeling parts, Tensorflow v. 3.8 [52] and its Keras extension were applied. Although multiple hardware platforms were used for different applications, all of them were desktop or laptop like computers with minimum RAM equal to 16 GB, 500 GB hard disk space, a CPU of equal or higher capacity than i7-4771 3.50 GHz running on Windows 10 or higher.

### 4.2. Databases

As speech and audio signals are very complex, it is necessary to have huge and specific datasets. In our experiments involving 4 different applications, we used 4 datasets to meet their specific properties.

#### 4.2.1. Speech Emotion Database

For SER-related experiments, we selected the Berlin database of emotional speech [53]. This database is in German and consists of 535 utterances that were sampled with a 16 kHz sampling frequency. Five men and five women (all actors) recorded 10 different texts in the studio environment. The recorded texts were not related to 7 supported emotional categories (anger, boredom, disgust, fear, happiness, neutral, sad). Fifteen people divided the recordings into categories based on their subjective perception.

#### 4.2.2. Speaker Recognition Database

In the speaker recognition task, we decided to use the LibriSpeech [54] dataset, which is open source and offers several subsets. We chose the Train-clean-100 subset having approx. 100 h of recorded speech comprising 251 different speakers (125 women, 126 men) who read different audio books in a “clean” environment. Each speaker recorded on average 25 min of eligible speech that was segmented into recordings. The recordings were of different lengths ranging from 2 to 40 s. The recordings were sampled with a 16 kHz sampling frequency using a 16 bit quantization.

#### 4.2.3. Speech Recognition Database

Since we aimed to test basic physical (acoustic) characteristics such as frequency, time and quantization, it was not necessary to incorporate a specific language model. Therefore, an isolated word recognition system covering a wide range of speakers and conditions, containing acoustically rich samples, would be enough. For this purpose, the Speech Commands Dataset v0.01 [55] was chosen. It contains 30 different words (commands) uttered multiple times by most of the participants. Altogether there are 58,000 1 s-long records produced by thousands of speakers. The database also contains both real and synthesized noises. The recordings were made in rather uncontrolled but interior environments all over the world. The recordings were acquired in different formats (using compression techniques as well), which at the end were converted to 16 bit, 16 kHz PCM samples. It should be noted that recordings shorter than 1s were not considered for further processing as it was observed that many shorter recordings contain only noise, although being labeled as true words.

#### 4.2.4. Audio Events Database

In our AER experiments, we opted for the ESC-50 dataset [56] for several reasons. It comprises a wide variability of sounds categorized into 5 broad classes, i.e., animals, nature and water, human (not speech), domestic, and city noise, each having 10 subclasses forming in total 50 distinct classes. Each class contains 40 recordings, which results in 2000 recordings. Each record lasts 5 s and contains only a single event with some background noise, allowing us to apply block-wise processing. The recordings are stored as PCM samples quantized in 16 bits with a sampling frequency of 44.1 kHz. It should be noted that for the feasibility (execution time), only 8 distinct classes were considered as follows: barking dog, fire, running water, crying baby, door knocking, glass smashing, firework, and sirens.

### 4.3. Networks and Training

In most of the experiments, single networks were used over the tested domains. If not stated otherwise, the networks are as follows. In case of SER tests, a network having 2 convolutional layers with 16 and 32 kernels of the size of 5 × 5, 2 max pooling layers of the size 2 × 2, a dense layer (128 neurones), a dropout layer, and a softmax layer having in total approx. 170k parameters was used. In speaker recognition experiments, a 5-layer CNN (3 convolutional and 2 dense layers) with 96 kernels of 2 × 2 sizes having approx. 50k parameters was used. For the speech recognition application, we deployed a 6-layer network (4 convolutional and 2 dense layers), with 40 kernels, 3 max pooling layers, and a single dropout layer that had in total approx. 55k parameters. Finally, in the AER tests, a 6-layer network (3 convolutional and 3 dense) with 168 kernels of 3 × 3 size, and 2 max pooling layers having in total approx. 1 M parameters was used.

The ADAM training algorithm was chosen to train the networks on datasets that were divided into training (80% of data), test (20% of data), and validation (20% of training data) sets, each having the same number of samples per class. An early stopping criterion was applied to prevent an overtraining.

## 5. Results

The experiments addressed following issues: frequency ranges, time (duration) aspects, quantization of signals, selection of vocal tract features, and the model complexity for speech, speaker, speech emotion, and audio event recognition. Thus, they were divided into 5 sections each having 4 subsections dedicated to a particular domain.

### 5.1. Time and Segmentation

This set of experiments was used to test speech/audio segmentation settings, i.e., lengths of frames (windows), their shifts, as well as the lengths of spectrograms and their shifts. These parameters control the time—frequency resolution, number of available data, size of FVs, and the degree of redundancy. Thus, they are directly related to memory requirements, processing delays, and a computational load.

#### 5.1.1. Emotion Recognition

**Figure 3:**
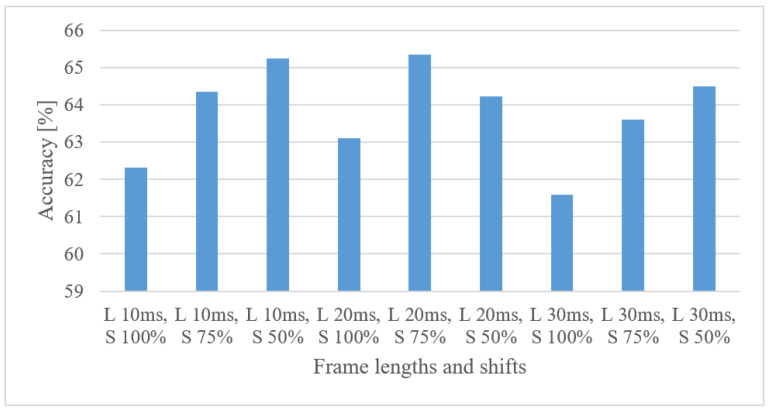
Averaged accuracies for 10, 20, 30 ms frame lengths (L) with 100, 75, 50% shifts (S) and a 0–8 kHz power spectrogram.

In Figure 3, accuracies for 10, 20, and 30 ms-long frames with 100, 75, and 50% shifts between frames using a 0–8 kHz power spectrogram are shown.

In all but one case, shorter shifts between frames resulted in higher accuracy. This suggests that the higher number of frames (even more redundant) in spectrograms is more beneficial in the use of CNNs. Frame lengths of 30 ms provided slightly worse results, indicating that the proper time–frequency resolution is provided by 10 to 20 ms-long frames.

**Figure 4:**
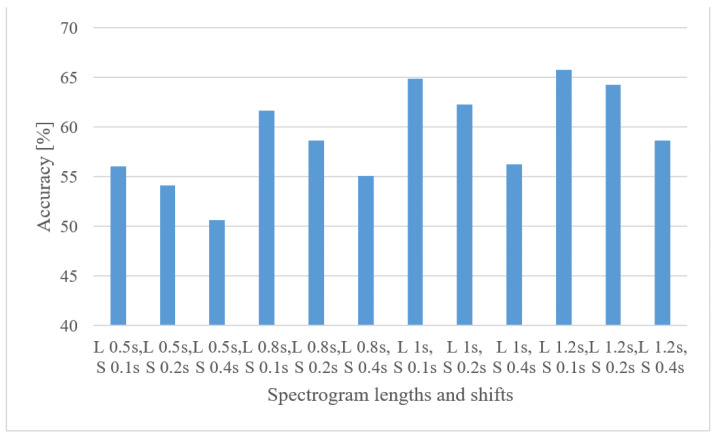
Averaged accuracies for spectrogram lengths (L) of 0.5, 0.8, 1, 1.2 s and 0.1, 0.2, 0.4 s shifts (S) between adjacent intervals.

Next, the time span of spectrograms and their time shifts were tested. The averaged accuracies for 0.5, 0.8, 1, 1.2 s-long spectrograms and 0.1, 0.2, 0.4 s-long shifts are shown in Figure 4 (the 1.2 s upper limit is given by the shortest recording).

As naturally expected, the longer speech intervals had higher accuracies. However, the increase started to level up above 1 s. Finally, the longer shifts resulted in worse accuracies, meaning the amount of training data is more important than the increased redundancy between FVs.

#### 5.1.2. Speaker Recognition

**Figure 5:**
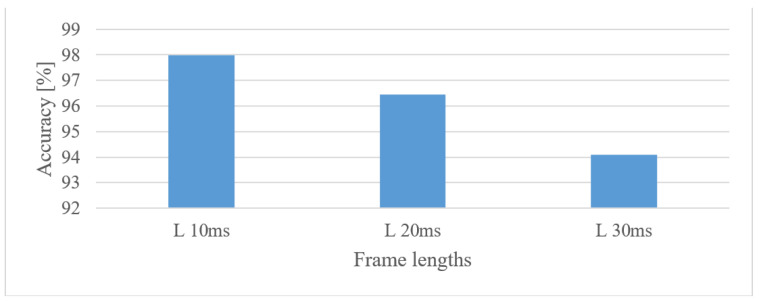
Averaged accuracies for 10, 20, 30 ms frame lengths (L) and 50% shifts for 1 s-long 0–10 kHz spectrograms.

In speech segmentation tests, 10, 20, and 30 ms-long frames with 50% shifts were tested. Their performance is shown in Figure 5 for 1 s-long 0–8 kHz spectrograms.

**Figure 6:**
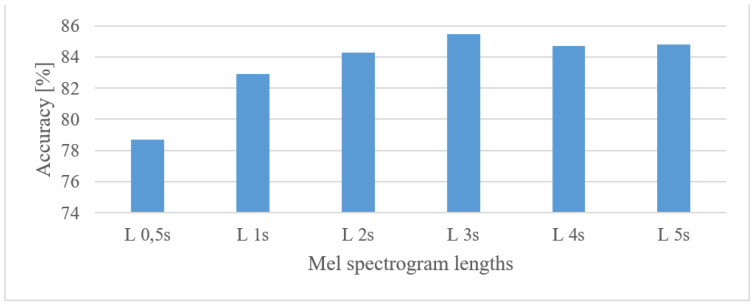
Accuracies of the measured lengths (L) of 100 Hz–10 kHz MFBs.

It is apparent that the shorter frames (higher time resolutions) scored better. This is partly attributed to the higher number of frames per spectrogram and thus longer FVs. In the time span test, Mel spectrograms (0.1–8 kHz) of the following durations were tested: 0.5, 1, 2, 3, 4, and 5 s; the results are shown in Figure 6.

As expected, there was an “optimal” time span of the analyzed speech, requiring a trade-off between the analyzed data and the number of training samples, i.e., the 3 s lengths of the analyzed speech seemed to be eligible.

#### 5.1.3. Speech Recognition

**Figure 7:**
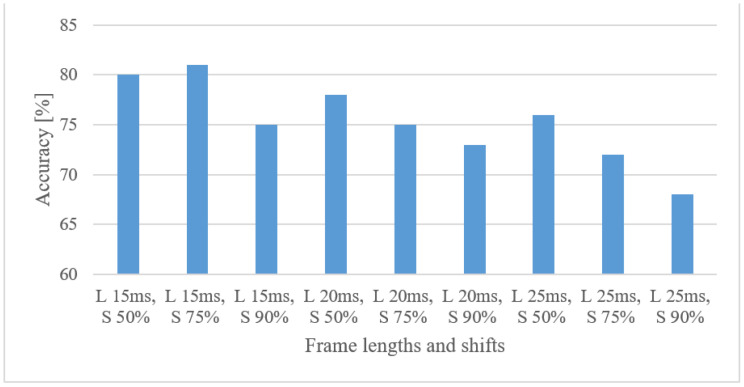
Accuracies for 15, 20, 25 ms frame lengths (L) and 50, 75, 90% shifts (S) between adjacent frames in the case of log spectrograms.

The results for 15, 20, and 25 ms frame lengths and 90, 75, and 50% shifts between adjacent frames are shown in Figure 7 for log spectrograms.

Shorter frames, e.g., 15 ms, slightly outperformed the longer ones, prioritizing spectrograms with more frames over a finer frequency resolution. Moreover, shorter shifts were preferred, which further increased the number of usable frames at the expense of redundancy.

#### 5.1.4. Audio Event Recognition

**Figure 8:**
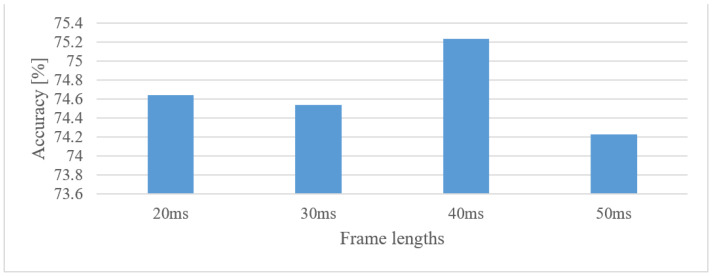
Averaged accuracies for 20, 30, 40, and 50 ms frame lengths in the case of 0–22,050 Hz spectrograms.

In case of general environmental sounds having unknown properties (in time and frequency), some processing parameters were tested in ranges that are uncommon in speech signals. The results for 20, 30, 40, and 50 ms frame lengths with a 50% shift are shown in Figure 8.

First, the differences in accuracy were rather insignificant across the tested frame length range (20–50 ms), i.e., the accuracy is not very sensitive to this parameter. Second, except for the 40 ms frame, a slightly declining trend in the accuracy can be spotted, suggesting that the standard speech frame length (20 ms) is suitable as well. The 40 ms outlier can most likely be attributed to a random nature of training (5-fold validation), as there was no obvious physical reason for this to happen, except that there are very special classes of sounds that require just this time–frequency resolution. Even so, this would not allow any generalization. Next, the eligible signal lengths of 500, 750, 1000, and 1500 ms and their 100, 75, 50 and 25% shifts were tested. For a better representation and because of a lack of space, these are listed in Table 1, together with averaged accuracies over signal lengths and shifts separately, to see potential trends. The best options are in bold.

It can be seen that the shorter shifts, i.e., more data (redundancy) are beneficial, whereas the optimal length of analyzed signal is somewhere between 750 and 1000 ms. This is a trade-off between the ability to capture a whole event and not interfere with another one or background noise. Thus, this may be affected by the set of events to be recognized.

### 5.2. Frequency Ranges

Frequency ranges are important parameters, as they may severely affect the recognition rates, amount of processed and stored data, quality, and cost of audio input sensors, etc. Moreover, they may differ among applications.

#### 5.2.1. Emotion Recognition

**Figure 9:**
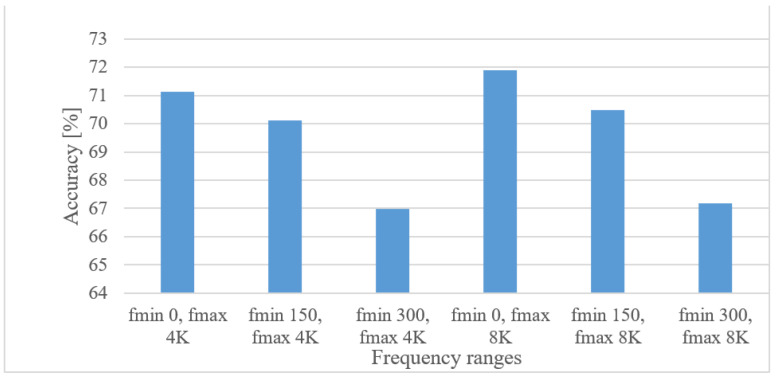
Averaged accuracies for minimal and maximal frequency limits.

The following options were examined: 0, 150, and 300 Hz as minimal frequencies and 4, and 8 kHz as maximal frequency limits. The averaged accuracies for lower and upper frequency limits are shown in Figure 9 for magnitude-based spectrograms. The positive effect of low frequencies is rather obvious for both upper limits. The 8 kHz upper frequency scored better in all cases; however, the increase was not significant.

#### 5.2.2. Speaker Recognition

**Figure 10:**
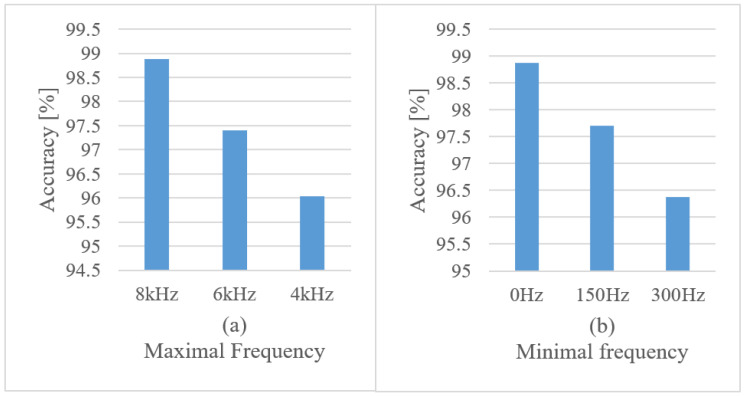
Accuracies for maximal frequency limits with the minimal frequency set to 0 Hz (**a**), and a minimal frequency test with the maximal frequency set to 8 kHz (**b**) using 1 s-long spectrograms.

In Figure 10a the effect of maximal frequency limits is shown for a fixed minimal frequency (0 Hz), and in Figure 10b the minimal frequency limits are depicted, while the maximal frequency is set to 8 kHz; the used FVs are 1 s-long spectrograms.

The tests clearly show that both maximal upper and lower frequency limits are needed to secure the best recognition scores.

#### 5.2.3. Speech Recognition

**Figure 11:**
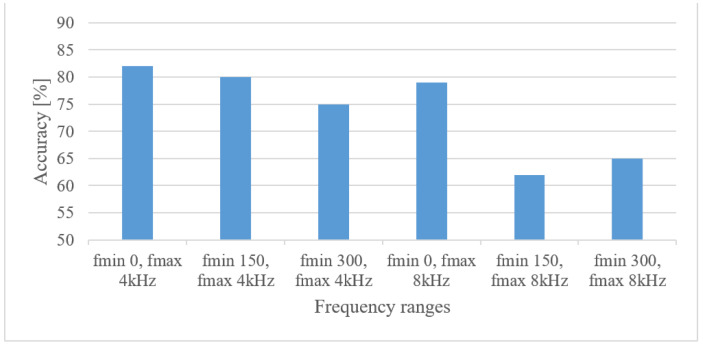
Accuracies for minimal and maximal frequency limits.

The results for 0, 150, and 300 Hz minimal frequencies combined with 4 and 8 kHz maximal frequencies are depicted in Figure 11 in the case of magnitude spectrograms.

From Figure 11 it is obvious that the 4 kHz frequency range is vital, whereas the merit of an 8 kHz upper frequency is rather limited, which is in line with a general knowledge (telephony, or intelligibility of speech). However, here even lower frequencies (below 300 Hz) recorded improvements.

#### 5.2.4. Audio Event Recognition

Since more frequencies for both upper and lower limits were tested, they are listed in Table 2 with averaged accuracies over lower and upper frequency limits so that the potential trends are more visible.

Based on the averaged values, the preferable upper and lower frequency limits were around 100 Hz to approx. 17.5 kHz with a limited potential to 22.05 kHz. However, the exact limits may depend on the specific sound classes that are to be separated.

### 5.3. Vocal Tract-Based Features: Filter Banks and Cepstral Coefficients

In addition to a basic representation of speech/audio signals by spectrograms, the more processed features focusing on vocal tract characteristics were examined. Here, the most common acoustic features, i.e., MFB and MFCC were tested to see if and to what extent they were beneficial with the use of CNNs.

#### 5.3.1. Emotion Recognition

**Figure 12:**
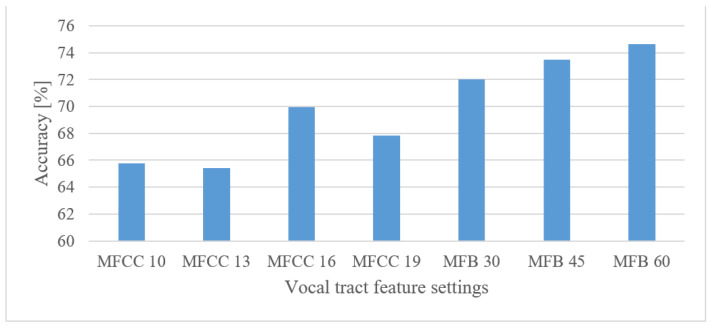
Averaged accuracies for 30, 45, and 60 MFBs, and 10, 13, 16, and 19 MFCC coefficients for an 8 kHz maximal frequency.

In Figure 12, the results for 30, 45, and 60 MFB, and 10, 13, 16, and 19 MFCC confinements are shown for an 8 kHz maximal frequency. It can be seen that the higher number of MFBs had a positive effect, whereas the eligible number of MFCC coefficients was around 16. When the best results are compared, MFBs scored better by approx. 3.6% than spectrograms. However, the MFCCs failed to achieve any improvement.

#### 5.3.2. Speaker Recognition

**Figure 13:**
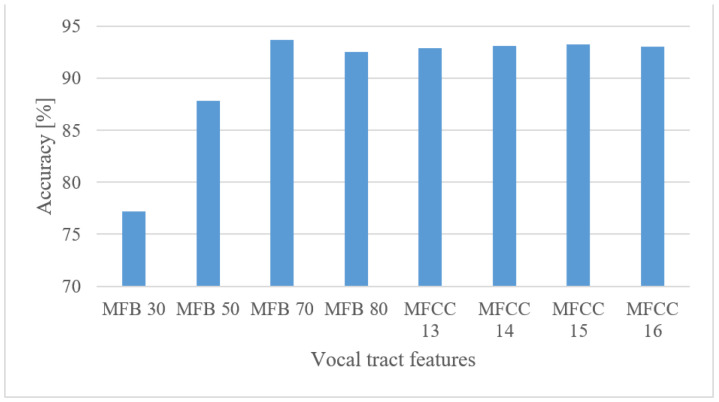
Accuracies for 30, 50, 70, and 80 Mel FBs, and 13, 14, 15, and 16 MFCCs for a frequency range of 0–8 kHz.

The performance of MFBs and MFCCs and their basic settings are shown in Figure 13.

There was a positive trend in the higher number of filter banks up to approx. 70. On the other hand, MFCCs produced more stable results over the tested range with a small peak at around 15 coefficients. This shows the ability of MFCCs to compress the acoustic information.

#### 5.3.3. Speech Recognition

**Figure 14:**
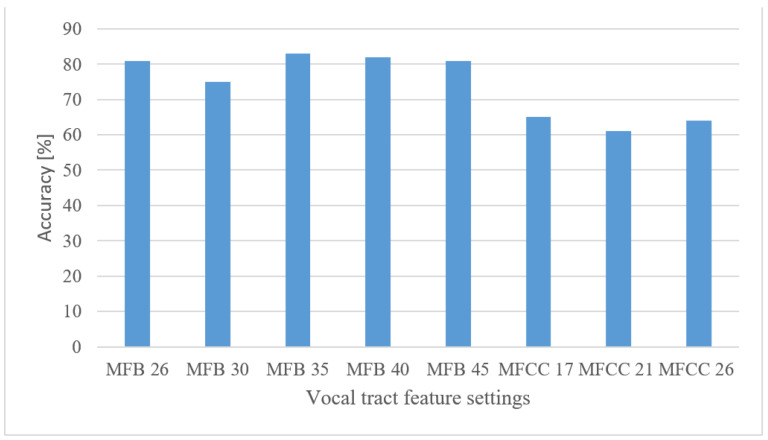
Accuracies for 26, 30, 35, 40, and 45 MFBs, and 17, 21, and 26 MFCC coefficients.

The results of vocal tract features are shown in Figure 14 for 26, 30, 35, 40, and 45 MFBs, and 17, 21, and 26 MFCCs. The numbers of MFCCs were 50, 60, and 75% of the 35 MFBs (best scorer) from which the MFCCs were calculated. The more processed features (MFCC) did not bring the desired improvements in combination with CNNs, suggesting that CNNs can still find a better representation. The appropriate number of MFBs was around 35, for which the MFBs slightly outperformed the magnitude-based spectrogram by approx. 1 percentage point.

#### 5.3.4. Audio Event Recognition

**Figure 15:**
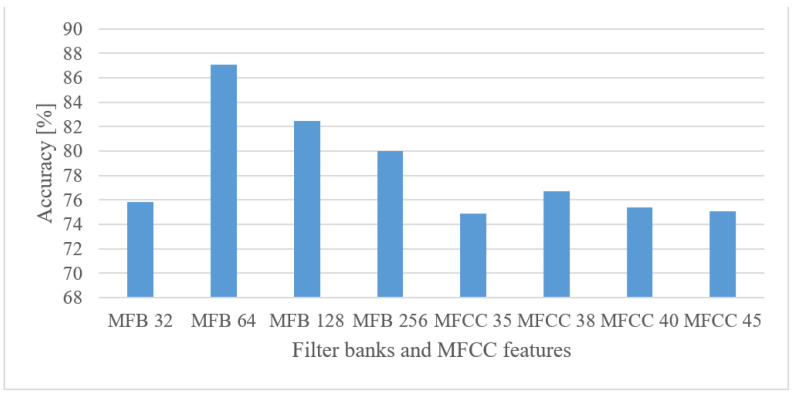
Accuracies for 32, 64, 128, and 256 Mel FBs, and 35, 38, 40, and 45 MFCC coefficients for a frequency range of 100 Hz–17.5 kHz.

The results for different settings of the MFB and MFCC features are depicted in Figure 15 for a 100 Hz–17.5 kHz frequency range.

In the case of MFBs there was a peak in the number of filter banks at around 64. Moreover, this feature slightly outperformed magnitude-based spectrograms. On the other hand, the more processed MFCC features did not record any improvements in combination with CNNs.

### 5.4. Quantization

In this set of experiments, the effect of precision of signal representation was tested. Across all applications, the following quantization schemes were used: 16 bit linear (original representation), 12 bit linear, 8 bit linear, and 8 bit µ-law (nonlinear). It should be noted that before quantization, signals were normalized to the interval (−1, 1).

#### 5.4.1. Emotion Recognition

**Figure 16:**
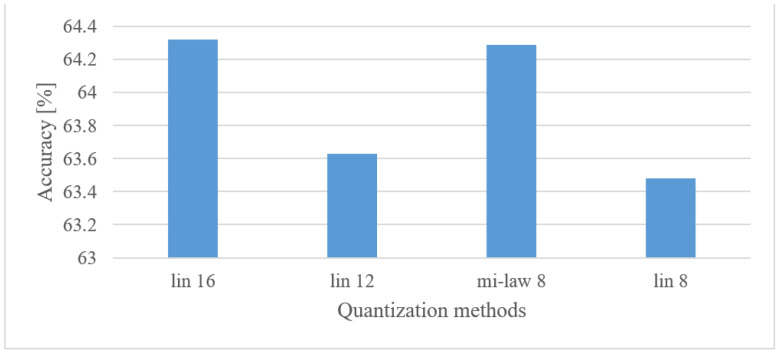
Averaged accuracies for 16, 12, and 8 bit linear, and 8 bit µ-law quantization schemes in the case of 1 s-long spectrograms.

For an SER system and 1 s-long spectrograms, the results obtained are shown in Figure 16. Although the original signal recorded the best score, the differences are not significant, especially in the case of 8 bit μ-law quantization. This indicates the performance was affected only in a minimal way, thus a significant amount of data can be saved in this application.

#### 5.4.2. Speaker Recognition

**Figure 17:**
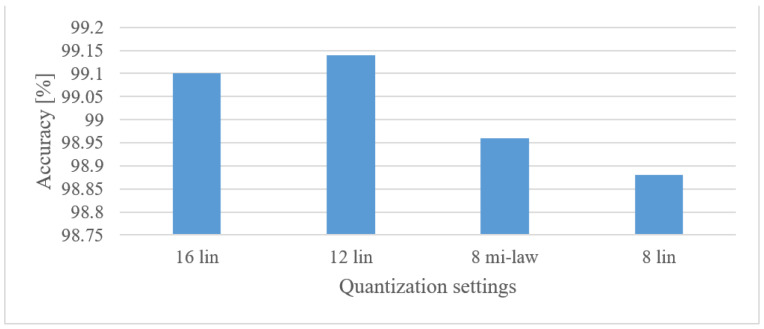
Accuracies for 16, 12, and 8 bit linear, and 8 bit µ-law quantization schemes for 0–10 kHz spectrograms.

Results for the tested quantization schemes are shown in Figure 17 for 1 s-long spectrograms covering a frequency range of 0–8 kHz.

Interestingly enough, and again, the effect of quantization was rather negligible in the tested range; nevertheless, the 8 bit representations provided slightly worse results.

#### 5.4.3. Speech Recognition

**Figure 18:**
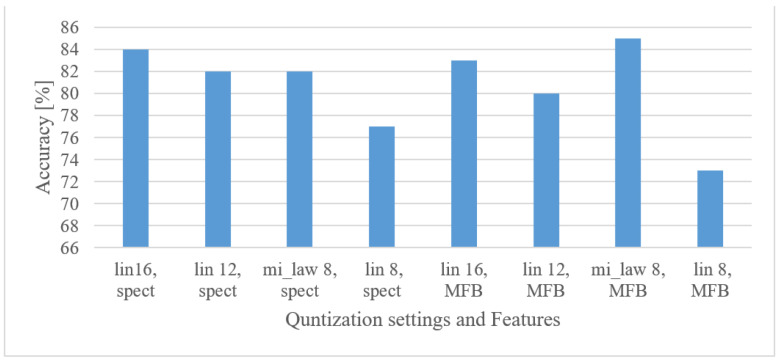
Accuracies for 16, 12, and 8 bit linear, and 8 bit µ-law quantization schemes in the case of spectrograms (spect) and MFBs.

The effect of quantization was tested using both spectrograms and MFBs as documented in Figure 18. It can be seen that the 12 bit linear and 8 bit µ-law quantization methods still provide competitive results, while saving significant space because of coarser representation. Moreover, this observation holds both for spectrograms as well as MFBs. On the other hand, the 8 bit linear quantization proved to be insufficient as it resulted in 7.6% (spectrogram) and 12% (MFB) drops in the accuracy.

#### 5.4.4. Audio Event Recognition

**Figure 19:**
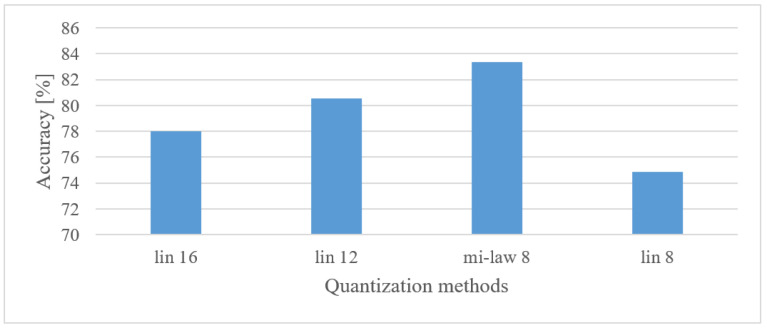
Accuracies for 16, 12, and 8 bit linear, and 8 bit µ-law quantization schemes in the case of 1 s-long spectrograms covering a range of 100 Hz–17.5 kHz.

Figure 19 shows the performance of an AER system using the tested quantization schemes.

As already observed, even severe quantization schemes did not necessarily lead to dramatic drops in the accuracy of the tested speech domains. Here, however, even improvements were observed for 12 bit linear and 8 bit µ-law schemes. This rather surprising result will be further discussed in the discussion section. Nevertheless, an application of coarser quantization, e.g., 8 bit µ-law, is possible and without negative effects on the accuracy.

### 5.5. Modeling Considerations

So far, a single CNN model was used across all experiments for a particular application. Such models were designed based on already published articles and customized using limited data. Next, for the best settings (features), the effect of model complexity is evaluated by testing additional models. This may be important for the hardware requirements, especially in the case of standalone applications with limited resources.

#### 5.5.1. Emotion Recognition

**Figure 20:**
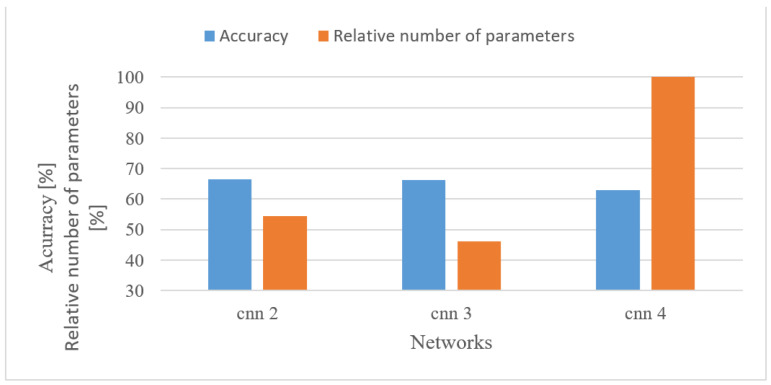
Averaged accuracies and relative numbers of trainable parameters for custom 2, 3, and 4 convolutional layer CNNs (CNN 2, CNN 3, CNN 4), and 0–8 kHz spectrograms.

The complexity of the model for SER systems was tested using three custom-designed networks. The first was the original marked as CNN 2 defined in Section 4.3. The second is CNN 3, which was a 5-layer CNN network having 3 convolutional and 2 dense layers with 3 max pooling layers, 112 kernels of 5 × 5 sizes and approx. 150k parameters. Finally, CNN 4 was a 6-layer CNN network having 4 convolutional and 2 dense layers with 4 max pooling layers, 240 kernels of 5 × 5 sizes, and approx. 310k parameters. The results for 1 s-long spectrograms (0–8 kHz) are shown in Figure 20 together with the relative number of trainable parameters that were related to the most complex network (CNN 4).

From Figure 20, it can be seen that the increased number of layers was not very beneficial, and even a CNN with two convolutional layers provided best results among the tested networks.

#### 5.5.2. Speaker Recognition

**Figure 21:**
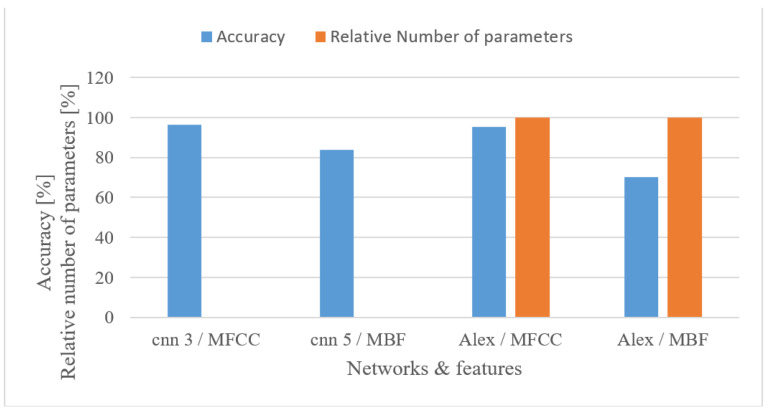
Accuracies and relative numbers of trainable parameters for 3- and 5-layer custom CNNs (CNN 3, CNN 5), and the Alexnet for MFCC and MFB features.

In the speaker recognition test, a hyper parameter tuning tool in the Keras system was deployed to find the “optimal” structure of a CNN for the features used (MFBs and MFCCs separately). The system was instructed to search for 3 to 6 convolutional layers, each with 16, 20, 32, 48, and 64 kernels of 2 × 2, 3 × 3, 4 × 4 shapes, and pooling layers. The best network for MFB had 5 convolutional layers with 20, 32, 64, 32, and 20 kernels of 2 × 2 sizes, 5 max pooling 2 × 2 layers, and a single dense layer with 251 neurons, i.e., in total approx. 39k parameters. In the case of MFCC, a 3-layer network with 32, 48, and 64 kernels of 3 × 3, 3 × 3, and 2 × 2 sizes, having the same number and sizes of pooling layers, and 2 dense layers with 208, and 251 neurons, having in total approx. 59k parameters, was found. For a complex network, the Alexnet [49] was selected for both features. The results for tested features and networks in terms of accuracy and relative number of trainable parameters that related to the most complex network (Alexnet 60 M parameters) are shown in Figure 21. As can be seen, MFCC features provided better results for both networks, and the optimized small-size networks slightly outperformed the complex pre-trained Alexnet. Moreover, the custom networks required only a small portion of trainable parameters, i.e., 0.098% and 0.047% of what Alexnet used.

#### 5.5.3. Speech Recognition

**Figure 22:**
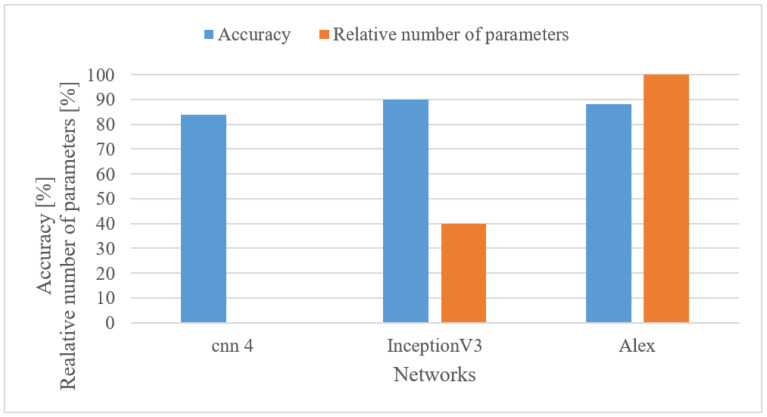
Accuracies and relative numbers of trainable parameters for a custom 4-layer CNN (CNN 4), Inception V3, and Alexnet networks and 0–4 kHz log spectrograms.

In addition to the custom-designed 4-layer CNN, pre-trained complex Inception V3 [50] and Alexnet networks were tested as well. Both were fine-trained using 1 s-long log spectrograms. In Figure 22, accuracies and the relative number of trainable parameters are shown; the parameters are relative to the maximal number of parameters (Alexnet).

Although the achieved accuracies differed slightly, ranging from 84 to 90%, the complexities were of huge contrast, e.g., 0.091% (CNN 4), 39.9% (Inception V3) and 100% (Alexnet). The best results were achieved with Inception V3 although it only had approx. 40% of Alexnet’s trainable parameters. This shows that the network structure is also important, and a relatively simple network can provide competitive results as well. Finally, it should be noted that the outcome may differ according to the size of the supported vocabulary.

#### 5.5.4. Audio Event Recognition

**Figure 23:**
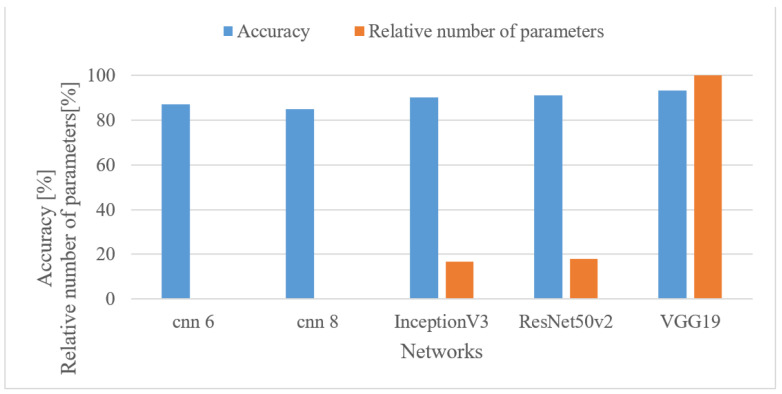
Accuracies and relative numbers of trainable parameters for custom 6- and 8-layer CNNs (CNN 6, CNN 8), Inception V3, VGG19, and ResNet50v2, and 100 Hz-17.5 kHz 64 MFBs.

In this experiment, several small-size custom-designed models were tested as well as pre-trained networks, i.e., Inception V3 [50], VGG19 [51], and ResNet50v2 [48]. The results, i.e., accuracies and relative number of free parameters, are shown in Figure 23 for 64 MFBs. Here, CNN 6 denotes a model with 6 convolutional layers, 4 dense layers, 3 max pooling layers, 224 kernels, and approx. 277k parameters, and CNN 8 represents a CNN with 8 convolutional layers, 4 dense layers, 4 max. pooling layers, 576 kernels, and approx. 198k parameters.

The accuracies were in a range of 85 to 93%, where the best result was provided by the most complex VGG19 network. The pre-trained models recorded better results than the custom small-size ones, i.e., by 7.3% (best cases). On the other hand, there were huge discrepancies among relative complexities, i.e., 0.19% (CNN 6), 0.18% (CNN 8), 16.6% (Inception), 17.8% (ResNet), and 100% (VGG19). This shows it is possible to use small-size models in case of limited resources at the expense of a slight decrease in the accuracy.

## 6. Discussion

To summarize the presented set of experiments and to make possible generalizations, the findings will be grouped according to domains.

Speech emotion recognition

- The best results were observed for a frequency range of 0 to 8 kHz, in line with expectations; however, using only a 4 kHz range caused only a small drop in the accuracy, i.e., by approx. 1%. On the other hand, the importance of low frequencies, i.e., less than 300 Hz, was more obvious, where more than a 6.5% decrease could be observed.
- In the case of time–frequency resolution, 20 ms-long windows provided the best results, whereas shorter shifts between adjacent frames were preferred. This resulted in more training data that were, on the other hand, more redundant. The recognition accuracy naturally grew with the length of analyzed speech intervals; however, the increase became saturated over the 1 s interval.
- The quantization was not relevant to the accuracy; i.e., all the tested settings provided rather similar results, e.g., in the worst case (8 bit linear) the decrease in recognition rate was only 1.3%, but using only half of the original precision.
- The representation of speech using MFBs (60) brought a 3.8% improvement compared to basic spectrograms while saving FV size. Thus, MFBs in combination with CNNs present more suitable FVs than spectrograms.
- Quite similar results were observed across CNNs of different complexities, i.e., 2, 3, and 4-layer CNNs with approx. 170 k, 145 k, and 310 k of free parameters varied at most by 5.2%, with CNN 2 being the best scorer. This suggests that even a CNN having 2 convolutional layers with approx. 150 k parameters fits such an application, while providing competitive results. This is in line with the findings in [7] showing that the deployment of complex pre-trained networks was of limited benefits.

Speaker recognition

- The best performance was observed in the 0–8 kHz frequency band, which provides valuable extra information for the speaker recognition task. If, for any reason, only a 4 kHz range is available, then more than a 3% drop in the accuracy can be anticipated. Lower frequencies (under 300 Hz) proved to be important as they could increase the accuracy by more than 2.8%.
- When speech frames are regarded, 10 ms-long windows recorded the best scores. This may suggest that the time structure (resolution) was more emphasized. However, this may be partly attributed to the existence of more data as there were more frames. Using shorter windows (10 ms) than the common 20 ms ones increased the accuracy by more than 1.5%. Furthermore, increasing the analyzed speech intervals steadily improved the recognition accuracy up to a duration of 3 s. After this the accuracy saturated and longer intervals, e.g., 4 or 5 s, increased FV sizes and delays more than the accuracy.
- The tested quantization schemes (ranging from 16 to 8 bits) had no significant effect on the accuracy, and the recorded variations can be more attributed to the stochastic nature of the training process, i.e., there was only a 0.2% difference in the accuracy when comparing the best (12 bit linear) and the worst (8 bit linear) cases.
- The vocal tract features represented by MFB and MFCC did not improve the results obtained by spectrograms. In fact, more than a 5.4% decrease in the accuracy was observed in the best settings. Spectrograms preserve some information about excitation signals, e.g., fundamental frequency, and that may be valuable for speaker recognition as well. On the other hand, vocal tract features, especially MFCCs, reduced the FV size.
- The effect of complexity was tested using 2 custom but still optimized CNNs (CNN3, CNN5) and the Alexnet for MFB and MFCC features. In both cases, the custom networks slightly outperformed the Alexnet (Figure 21), while using only a fraction of free parameters, i.e., 0.098% (3-layer CNN) and 0.065% (5-layer CNN). This suggests that even low-complexity networks having 3 to 5 layers and approx. 60 k parameters can achieve outstanding results.

Speech recognition

- The best performance was provided by a frequency range of 0 to 4 kHz for both magnitude-based and log spectrograms. This is not surprising as such a band is a standard for speech recognition. The 8 kHz band caused a decrease in the accuracy for all tested cases, and moreover, it somehow disrupted the otherwise homogeneous positive effect of low frequencies, especially in the case of log spectrograms. This may indicate that outside this common range there is mostly non-lexical information and noise that is even emphasized by a log function.
- When a time–frequency resolution is regarded (Figure 7), 15 ms-long windows provided the best results, as well as the shorter shifts between adjacent frames. Both parameters resulted in more training data, which were, however, more redundant. Furthermore, shorter windows prioritized time resolution over frequency, which seemed to be beneficial in combination with CNNs.
- Quantization was tested both for spectrograms and MFBs. Here, the adverse effect of low bit representation, i.e., 8 bit linear, was the most apparent of all applications. Using an 8 bit linear quantization led to 7.6% (spectrogram) and 12% (MFB) degradations in the recognition rate. However, the less severe quantization schemes (12 bit and 8 bit μ-law) performed much better. This indicates that the lexical information encoded in speech signals is more sensitive to the degradation caused by quantization.
- Properly set MFBs, i.e., having 35–45 banks, provided almost similar results compared to basic spectrograms, while using shorter FVs. However, the more compressed MFCCs features failed in combination with CNNs as they scored approx. 23% worse than spectrograms.
- The more complex and pre-trained networks (Alexnet and Inception V3) achieved better results than the custom-designed 4-layer CNN having only approx. 55 k parameters. There was a 7.1% increase in accuracy; however, the custom network used only 0.22% of free parameters of the Inception V3 network. Nevertheless, this gives designers an option of making a trade-off between accuracy and complexity.

Audio event recognition

- By averaging the results over lower and upper frequency limits separately, the reasonable band was approx. 100 Hz–17.5 kHz. This means that many events in the environment have significant information located also in higher frequencies. If only limited band, e.g., up to 4 kHz is assumed, one can expect approx. a 12% decrease in the accuracy.
- In the frame length test (Figure 8) the results showed a high degree of insensitivity to the tested sizes (20–50 ms). In spite of an outlier, there seems to be a trend of preferring shorter windows, e.g., 20 ms ones similar to speech processing applications. When the lengths of analyzed time intervals are considered, the best results were observed in the range from 0.75 to 1 s.
- Quantization tests (Figure 19) provided rather unexpected results. No matter how weird these may look, they can still have explanations other than just the “random nature” of the training. We suspect that the following phenomenon may have taken place here. First, the quantization may have suppressed or normalized some noise (background) that deteriorated the recognition process. This is because high-frequency components (e.g., over 8 kHz) usually tend to rapidly vanish for common signals, meaning that such components are most of the time rather noisy, and by introducing coarser quantization, such noise can be partly suppressed or normalized. Next, the quantization noise may have masked portions of signals that were confusing in the classification stage for most similar classes, or the quantization may have reduced variability for some classes of signals, i.e., introducing some sort of normalization, etc. However, a more credible explanation of this phenomenon requires a different and thorough investigation. Here, only the benefits of coarser quantization were measured and presented.
- MFBs (64) proved to be beneficial for AER as they slightly outperformed the basic representation by spectrograms, while reducing the FV size. On the other hand, the more compact features of MFCC failed, as they caused approx. a 12% decrease in the accuracy.
- The pre-trained complex models scored better than the custom small-size networks by 7.3% when the best cases were compared. However, when relative complexity is considered, the custom-designed network had only 0.19% of VGG19 parameters. Such a comparison urges a designer to make a proper trade-off between complexity and computational load and accuracy.

Nevertheless, the abovementioned findings can still carry some issues such as:

- Even though CNNs are a very complex classification method with outstanding results in image/speech processing, we cannot reject the fact that the findings here can be partly affected by their limitations (structure, training algorithm, numerical precisions, etc.).
- Some settings especially related to the model complexity are class size-dependent, i.e., the complexity may differ based on the number of classes these systems are to recognize. This may substantially vary for applications such as speech recognition (30 words were tested), speaker recognition (251 speakers), and audio events (8 events).
- Even though some datasets also contained noise (real environment), this may still be slightly different from a particular deployment scenario.

## 7. Conclusions

Several specific experiments focused on fundamental speech and audio signal properties were presented using well-known datasets and tools. Such experiments aimed to analyze whether and to what extent the quality and amount of acquired signals affect major speech/audio processing applications, such as speech, speaker, speech emotion, and audio event recognition. Therefore, such an analysis may help designers with properly choosing structure, hardware, input sensors, and software for their applications. An appropriate use of the presented results may reduce the overall cost and complexity of new systems, while preserving high performance. From the broad set of experiments, the input audio sensor relevant findings can be summarized as follows.

Applications that require broadest spectra are AER systems, where the highest performance is observed in the range of 100 to approx. 18 kHz. It is followed by speaker and speech emotion recognition systems, both covering approx. a 0–8 kHz frequency band. Finally, the highest performance of a speech recognition system (speaker-independent) was observed in the range of 0 to 4 kHz. An interesting observation is the relevance of lower frequencies, i.e., less than 300 Hz, for speech-related applications.

Three applications (speaker, emotion, and audio event recognition) proved to be rather robust against the tested quantization schemes, showing 12 bit linear and 8 bit μ-law methods performing equally, even sometimes better than a standard 16 bit linear quantization. This can simplify input audio sensors and reduce stored and transferred data. The most sensitive to quantization degradation was a speech recognition application, where the 8 bit linear method caused a 7.6% deterioration in case of spectrograms. Nevertheless, for finer quantization, this effect was not very substantial (2.4% degradation). Thus, the correct choice of this parameter proved to be quite important for speech recognition systems.

When time aspects are considered, speaker recognition systems required longest intervals of analyzed signals in order to achieve a high performance, i.e., approx. 3 s of eligible speech. On the other hand, the effective time span for SER systems is 1 to 1.5 s. The least demanding are AER systems, requiring from 0.75 to 1 s-long intervals. This knowledge gives an idea about the size of needed buffers and introduced time delays.

## Figures and Tables

**Table 1:** Effect of signal lengths and shifts on accuracy. Averaged values for lengths and shifts are also given. The best options are in bold.

Shifts [%]	Length 500 ms	Length 750 ms	Length 1000 ms	Length 1500 ms	Average [%]
Shift 100%	70.74	72.08	71.83	61.66	69.07
Shift 75%	70.12	69.27	74.3	70	70.92
Shift 50%	72.71	**75**	74.35	73.25	**73.82**
Shift 25%	72.9	74.09	73.92	72.24	73.28
Average	71.61	72.61	**73.6**	69.2875	-

**Table 2:** Accuracies for different lower- and upper-frequency limits. The averaged values for both limits are also given. The best options are in bold.

	Fmax 4 kHz	Fmax 8 kHz	Fmax 12.5 kHz	Fmax 17.5 kHz	Fmax 22.05 kHz	Average [%]
Fmin 0 Hz	75.72	79.61	81.22	79.61	81.55	79.5
Fmin 100 Hz	75.08	85.37	84.78	85.11	**85.76**	**83.2**
Fmin 200 Hz	75.72	82.52	71.52	80.25	81.87	78.34
Fmin 300 Hz	74.75	72.49	78.64	83.81	79.54	77.84
Average	75.91	79.99	79.04	**82.2**	82.18	-

## Data Availability

The datasets supporting the conclusions of this article are available at: http://emodb.bilderbar.info/docu/ (Berlin database of emotional speech, accessed on 1 July 2022), https://www.openslr.org/12 (LibriSpeech, accessed on 1 July 2022), https://github.com/karolpiczak/ESC-50 (ESC 50, accessed on 1 July 2022), https://pyroomacoustics.readthedocs.io/en/pypi-release/pyroomacoustics.datasets.google_speech_commands.html (Speech Commands Dataset, accessed on 1 July 2022). In a part of our experiments we used TensorFlow platform available at: https://www.tensorflow.org/install? (accessed on 10 June 2022) under Apache License 2.0, implemented in the Python 3.8 programming language.
